# Disease Produced by Drinking Water Flowing from a Graveyard

**Published:** 1884-08

**Authors:** L. G. Hardman

**Affiliations:** Harmony Grove, Ga.


### DISEASES PRODUCED BY DRINKING WATER FLOWING FROM A GRAVEYARD—HISTORY OF THE CASE, ETC.

BY L. G. HARDMAN, M. D., HARMONY GROVE, GA.

The following cases present some points I think worthy of serious consideration:

H., age 13, female of previous good health, while at work in a field on October 1st, 1883, just before taking her dinner drank water from a branch which had its origin from the side of a hill that had long been used as a burying-ground.

The place at which she drank the water was about one hundred and fifty yards from the source of the branch. About an hour after she had taken the water she complained of being sick, suffered pain in head and very soon began to vomit, and continued until midnight, when a diarrhœa set up, from which she suffered greatly, complaining of aching all over, which continued for three days, when I was sent for to see her.

I found her with pulse 112, temperature 103° F., suffering intense pain in left hip and leg, tenderness on pressure over the sciatic nerve on same side; some swelling of the hip.

I gave patient hypodermic injection of morphine, which gave some relief. The morphine had to be continued at intervals to produce rest.

On the third day after I saw her, her mother called my attention to a swelling of the labia majora of the left side. This very soon resulted in a large abscess which discharged profusely. A few days after this she complained of pain in the knee, wrist and clavicula articulations. These showed very little sign of inflammation at first, but in a few days suppurated. The parotid glands also suppurated. Patient died on the eighteenth day. The temperature and pulse varied very little from what they were when I first saw her. She was given quinine and iodine, and was kept on as nutritious a diet as could be had, consisting of beef, eggs, etc.

There was a sister who drank water at the same time and place who was sick in the same manner, who had a similar abscess on the scalp to the ones related. This patient recovered.

A year previous to this time an older sister, after drinking at this same place, was taken sick in a few hours and had a long and severe spell of typhoid fever; she had no abscesses as did the others.

The owner of the land through which this branch flows, informs me that whenever he has permitted his stock to pasture in this field they lose their appetite, fail to eat and very soon get poor. He, knowing the bad effects of this water on his stock, had cautioned his children against drinking it.

Did the drinking of this water produce these diseases? If so, why should two of the cases seemingly have pyæmia and the other typhoid fever?

